# Pathology and Genetics of Tumours of the Nervous System

**DOI:** 10.1054/bjoc.2000.1562

**Published:** 2001-01-01

**Authors:** Michael Brada

**Affiliations:** Reader and Consultant in Clinical Oncology, The Institute of Cancer Research, The Royal Marsden NHS Trust, Downs Road, Sutton, Surrey, SM2 5PT, UK


*British Journal of Cancer* (2001) **84**(1),148
© 2001 Cancer Research Campaign
doi: 10.1054/ bjoc.2000.1562, available online at http://www.idealibrary.com on IDEAL®

http://www.bjcancer.com

### Book review

### Pathology and Genetics of Tumours of the Nervous System

Paul Kleihues and Webster Cavenee, eds, pp 314,
IARC Press, Lyon 2000. Price £46.00. ISBN 92 832 24094

Reading the classification of tumours is not a favourite pastime for a busy clinician. Yet a full understanding of the evolving knowledge of tumour biology and pathology is important as it impacts on everyday oncological practice. Behind a conventional title implying a weighty textbook of pathology where classification is the main remit lies an impressive modern multi-author book which will become indispensable for anyone dealing with neuro-oncology. No longer is pathology just a descriptive specialty of macroscopic and microscopic pattern recognition. It is an integrated subject combining biological, pathological and clinical information.

The book edited by Paul Kleihues and Webster Cavenee is a summary of the latest WHO classification of nervous system tumours written jointly with and by the participants of the WHO classification meeting group. Although a multi-author text, each chapter devoted to a specific CNS tumour follows a uniform scheme of defined sections ranging from molecular and cellular to clinical aspects of all nervous system tumours. The clear structure makes for easy navigation through a comprehensive and broad text.

Each chapter starts with a pathological definition which occasionally strays into clinical description. This is followed by details of demographics, epidemiology and clinical presentation. The histopathology sections, certainly from a clinician's point of view, are comprehensive and fully illustrated. In keeping with the expansion of biological research in brain tumours there is a wealth of biological information with up-to-date and comprehensive sections on molecular genetics. Every new WHO classification clarifies disease entities which have come to be recognized in the intervening period since the previous classification. This book provides a clear description of central neurocytoma and the now fully recognized atypical teratoid/rhabdoid tumour, which contains rhabdoid cells and occasionally resembles PNET. The pathological description has been underpinned by the recognition that 90% of tumours demonstrate monosomy or deletion of chromosome 22, most likely involving the INI1 gene. Other uncommon tumours are also included.

The molecular and cytogenetics parts of the chapters on astrocytic tumours are examples of an in-depth review of a fast-evolving subject. They include details of all the known chromosomal defects, the concept of primary and secondary glioblastoma and a full description of the currently recognized molecular lesions of the cell cycle and signalling pathways. Unfortunately, the progress in biological understanding of glial tumours has not been matched by advances in the treatment of glial tumours – a cause of frustration to anyone looking after brain tumour patients. Appropriately, the authors refrain from providing too much information about therapy and reserve comments to predictive factors, which have to be therapy related. Nevertheless, a concise clinical précis, which would serve as an objective summary of current clinical strategies and controversies, would greatly enhance this book.

Increasing reliance on electronic publications is frequently considered as a death knell to anything on paper and to textbooks in particular. This publication is a proof that books are alive and well, especially when clearly laid out and fully illustrated. It is possible not only to home in on the desired information but also to browse, and for the student and expert to learn.

*Pathology and Genetics of Tumours of the Nervous System* started life as a mere pathological classification of tumours. It is now transformed into a comprehensive and easily accessible text which will undoubtedly become a standard reference book. If the editors will continue to maintain the up-to-date feel with latest information on biology, as they managed in this edition, this unassuming book will also become indispensable for anyone interested in the future of translational research.

*Dr Michael Brada*

*Reader and Consultant in Clinical Oncology*
*The Institute of Cancer Research*
*The Royal Marsden NHS Trust*
*Downs Road, Sutton*
*Surrey SM2 5PT, UK*

